# *Cordyceps militaris* Extract and Cordycepin Alleviate Oxidative Stress, Modulate Gut Microbiota and Ameliorate Intestinal Damage in LPS-Induced Piglets

**DOI:** 10.3390/antiox13040441

**Published:** 2024-04-08

**Authors:** Shijie Xiong, Jiajia Jiang, Fan Wan, Ding Tan, Haibo Zheng, Huiqin Xue, Yiqiong Hang, Yang Lu, Yong Su

**Affiliations:** 1 Institute of Animal Husbandry and Veterinary Science, Shanghai Academy of Agricultural Sciences, Shanghai 201106, China; 2022105066@stu.njau.edu.cn (S.X.); wanfan@saas.sh.cn (F.W.); xuehuiqin@saas.sh.cn (H.X.); hangyiqiong@saas.sh.cn (Y.H.); 2 Laboratory of Gastrointestinal Microbiology, Jiangsu Key Laboratory of Gastrointestinal Nutrition and Animal Health, College of Animal Science and Technology, Nanjing Agricultural University, Nanjing 210095, China; 2022105067@stu.njau.edu.cn (D.T.); 2022805116@stu.njau.edu.cn (H.Z.); 3 Institute of China Black Pig Industry Research, Zhejiang Qinglian Food Co., Ltd., Haiyan 314317, China; ql058@qinglianfood.com; 4 Shanghai Engineering Research Center of Breeding Pig, Shanghai 201106, China

**Keywords:** piglets, *Cordyceps militaris* extract, cordycepin, intestinal microbiota, intestinal immunity, oxidative stress

## Abstract

Cordycepin is considered a major bioactive component in *Cordyceps militaris* extract. This study was performed to evaluate the ameliorative effect of *Cordyceps militaris* extract (CME) and cordycepin (CPN) supplementation on intestinal damage in LPS-challenged piglets. The results showed that CPN or CME supplementation significantly increased the villus height (*p* < 0.01) and villus height/crypt depth ratio (*p* < 0.05) in the jejunum and ileum of piglets with LPS-induced intestinal inflammation. Meanwhile, CPN or CME supplementation alleviated oxidative stress and inflammatory responses by reducing the levels of MDA (*p* < 0.05) and pro-inflammatory cytokines in the serum. Additionally, supplementation with CPN or CME modulated the structure of the intestinal microbiota by enriching short-chain fatty acid-producing bacteria, and increased the level of butyrate (*p* < 0.05). The RNA-seq results demonstrated that CME or CPN altered the complement and coagulation-cascade-related genes (*p* < 0.05), including upregulating gene *KLKB1* while downregulating the genes *CFD*, *F2RL2*, *CFB*, *C4BPA*, *F7*, *C4BPB*, *CFH*, *C3* and *PROS1*, which regulate the complement activation involved in inflammatory and immune responses. Correlation analysis further demonstrated the potential relation between the gut microbiota and intestinal inflammation, oxidative stress, and butyrate in piglets. In conclusion, CPN or CME supplementation might inhibit LPS-induced inflammation and oxidative stress by modulating the intestinal microbiota and its metabolite butyrate in piglets.

## 1. Introduction

In recent years, there has been an increased focus on the intestinal health of piglets due to the rapid growth of the animal husbandry industry. The intestine is the primary digestive and absorptive site and is also the largest immune organ, playing an essential role in maintaining normal immune defense function [1]. Intestinal health plays a crucial role in piglet growth and development, immune function and disease resistance. Therefore, it is essential to preserve the integrity and function of the intestinal barrier to ensure intestinal health [2]. However, piglets can be affected by various environmental and nutritional factors during growth, particularly during the weaning stage [3]. These factors can lead to intestinal injury, inflammatory responses, diarrhea, and even death [2,4], resulting in significant economic losses to livestock production. Therefore, alleviating weaning stress and maintaining the intestinal structural and functional integrity is essential for piglet health and production. Previous studies have proved that intestinal microbes and metabolites establish strong metabolic/nutritional networks with the host that could inhibit the growth of potential pathogens [5]. Furthermore, disruptions to the microbial community of the intestines are closely linked to the breakdown of intestinal barrier function and an increase in inflammatory responses [6]. Therefore, regulating the intestinal microbiota in the early stages of growth may be an effective way to prevent intestinal damage and related diseases. Recently, natural nutritional supplements have been widely reported to play a role in preventing weaning stress [7], maintaining the barrier function of the intestinal mucosa and alleviating intestinal disorders [8,9].

*Cordyceps militaris* is an entomopathogenic fungus that is found exclusively in the Himalayas and contains various bioactive components such as adenosine, cordycepin, extracellular polysaccharide fractions, peptides, and alkaloids [10,11,12,13]. As one of the main active components in *Cordyceps militaris*, cordycepin has been used as a dietary supplement and pharmaceutical due to its multiple biological functions, including anti-oxidative, anti-inflammatory and immunomodulatory, in animals and humans [14,15,16]. An in vitro study demonstrated that cordycepin can lead to the remission of the inflammation response by reducing the levels of pro-inflammatory cytokines (IL-1β, IL-6 and TNF-α) in LPS-induced RAW264.7 macrophages [17]. Moreover, a recent study has showed that oral cordycepin could improve the inflammation response and ameliorate oxidative injury by modulating the intestinal microbiota in Western-diet-induced obese mice [8]. Similarly, *Cordyceps militaris* extract exhibits anti-inflammatory activity by reducing the production and expression of inflammatory mediators in DSS-induced mice with acute colitis [18]. *Cordyceps militaris* extract is effective in the alleviation of oxidative-stress-induced apoptosis and premature ageing, the inhibition of cancer cell proliferation and the treatment of metabolic disorders caused by obesity [19,20,21]. Although previous research has indicated that cordycepin and *Cordyceps militaris* extract could modulate the composition of the gut microbiota, the potential mechanisms by which *Cordyceps militaris* extract and cordycepin regulate the association between intestinal inflammation and the gut microbiota remain unknown.

LPS can induce intestinal immune stress and disrupt the microbial balance in the intestinal tract, leading to inflammatory responses, impaired immune function and poor animal health [22].Therefore, in this study, we hypothesized that *Cordyceps militaris* extract and cordycepin supplementation could improve the intestinal microbiota and its metabolites to reduce intestinal inflammation and oxidative injury. To test this hypothesis, a model of intestinal injury in piglets induced by lipopolysaccharide (LPS) was used to evaluate the effect of *Cordyceps militaris* extract and cordycepin supplementation on the intestinal microbiota and its metabolites and their role in alleviating intestinal inflammation. The results of this study will contribute to our understanding of the role of *Cordyceps militaris* extract and cordycepin in attenuating oxidative stress and inflammatory responses in the intestinal of piglets. Collectively, this study examined the effects of dietary *Cordyceps militaris* extract and cordycepin on oxidative stress, intestinal immunity and the intestinal microbiota in LPS-induced piglets to reveal the potential mechanisms by which CPN and CME regulate intestinal health.

## 2. Materials and Methods

### 2.1. Preparation of Cordyceps militaris Extract and Cordycepin

The *Cordyceps militarist* residues were purchased from Jiangsu Kangneng Biological Engineering Co., Ltd. (Yang Zhou, China). In this experiment, the *Cordyceps militaris* extract (CME) was prepared by the Shanghai Academy of Agricultural Sciences (Shanghai, China) and the cordycepin (CPN) was acquired from Shanghai Yuanye Bio-Technology Co., Ltd. (Shanghai, China) with a purity of 90%. The CME, containing 1% cordycepin, was prepared through a process of water extraction, solid–liquid separation, reduced-pressure concentration and spray-drying the residue from *Cordyceps militaris*. Subsequently, further purification and a drying treatment were employed to obtain CPN with a content of 90%.

### 2.2. Animals and Experimental Design

The animal study protocol was approved by the Animal Care and Use Committee of the Shanghai Academy of agricultural sciences (SAASPZ0522050) and implemented based on the standard of Experimental Animal Care and Use Guidelines of China (EACUGC2018-01). The experiment was conducted in Zhenjiang City, Jiangsu Province, China. A total of twenty-four healthy male weaned piglets (7.37 ± 0.52 kg body weight (BW); Duroc × Landrace × Large White) were randomly assigned to 4 groups (*n* = 6): (1) Control group (CON), (2) LPS group (LPS), (3) LPS + CPN group (CPN-LPS), and (4) LPS + CME group (CME-LPS). The CON and LPS groups were provided with basal diets, while the CME-LPS and CPN-LPS groups were given basal diets supplemented with CME or CPN to achieve a final feed concentration of 1 mg/kg cordycepin. The experiment lasted for 21 days, with a 3-day adaptation period. The basal diet (Table 1) was formulated to meet the nutrient requirements for piglets as recommended by the NRC 2012 [23]. All piglets (single pen feeding) had ad libitum access to feed and water throughout the experiment. Feeding occurred three times per day at 7:00, 12:00, and 17:00.

### 2.3. LPS Injection

Similar to a previous study [24], on the 21st day, piglets in the LPS, CPN-LPS, and CME-LPS groups received intraperitoneal injections of LPS (100 μg/kg of BW, *Escherichia coli* O55:B5, Sigma Chemical Inc, St. Louis, MO, USA), while piglets in the CON group received the same volume of saline. The piglets were fasted prior to slaughter.

### 2.4. Sample Collection

All six piglets in each group were sacrificed four hours after LPS or saline injection under pentobarbital sodium anesthesia. Blood and intestinal samples were collected four hours after the injection of LPS or saline in this experiment. All animal samples were collected within two hours. Blood samples (10 mL) were collected from the anterior vena cava in sterile vacuum tubes and centrifuged at 3000× *g* for 10 min at 4 °C to obtain serum. The abdominal cavity was then dissected, and the small intestine was separated from the mesentery and placed at low temperatures. The ileum digesta sample was collected for microbial analysis. Mucosal samples from the mid-jejunum and mid-ileum were gently scraped with sterile slides after rinsing with ice-cold saline, immediately snap frozen with liquid nitrogen and stored at −80 °C for further analysis. Approximately 2 cm of tissue was cut from the middle of the jejunum and ileum, gently washed with normal saline, and stored in a 4% paraformaldehyde solution for analysis of the intestinal morphological indicators.

### 2.5. Growth Performance

The fasting weight of the piglets was measured at 6 am on the 1st and 21st days of the experiment and the daily feed intake per pen was recorded to calculate the average daily feed intake (ADFI), average daily gain (ADG) and feed-to-weight ratio (F/G).

### 2.6. Intestinal Morphology

The samples of intestinal segments were removed after fixation in 4% paraformaldehyde for 24 h, and the fixed intestinal segments were subjected to a series of fractional ethanol dehydration treatments, then degreased by xylene treatment, and embedded in paraffin. Sections of 4 μm thickness were stained with haematoxylin–eosin (HE). The stained slides were observed under the ECLIPSE E100 microscope (Nikon Instruments Inc., Shanghai, China). The villus height and crypt depth of six randomly selected villi from different sections were quantified using ImageJ software (Version 1.53, National Institutes of Health, Bethesda, MD, USA).

### 2.7. Serum Antioxidant Indices

Serum antioxidants, the total antioxidant capacity (T-AOC), total superoxide dismutase (T-SOD), malondialdehyde (MDA), catalase (CAT) and glutathione peroxidase (GSH-Px) were measured using biochemical assay kits (Jiancheng Biochemical, Nanjing, China).

### 2.8. Serum and Ileum Cytokine Levels

The intestinal mucosa samples were removed from the refrigerator at −80 °C and ground to powder using liquid nitrogen. They were weighed (0.1 g) and homogenized with ice-cold PBS solution (5 min), vortexed for 30 s, and then centrifuged at 4 °C for 12,000× *g* (10 min) to obtain the supernatant for the intestinal cytokine assay. The protein concentration was determined by the bicinchoninic acid (BCA) assay method with the protein detection kit (Biosharp Life Science, Hefei, China). According to the manufacturer’s instructions (Enzyme-Linked Biotechnology, Shanghai, China), the interleukin-1β (IL-1β), interleukin-6 (IL-6), interleukin-8 (IL-8), interleukin-10 (IL-10) and tumor necrosis factor-α (TNF-α) levels in serum and ileum were determined using ELISA kits. All procedures of cytokine determination were performed according to the manufacturer’s instructions.

### 2.9. 16SrRNA Analysis of Ileal Chyme Microbiota

The DNA of the ileal digesta was extracted with a DNA extraction kit (Axygen Biosciences, Union City, CA, USA), as provided by the manufacturer. Under the same conditions (95 °C for 2 min, followed by 27 cycles at 95 °C for 30 s, 55 °C for 30 s, 72 °C for 60 s and a final extension at 72 °C for 5 min), the V4–V5 region of the 16S rRNA gene was amplified with the primers 515F and 907R. Amplicons were extracted from 2% agarose gels and purified using the AxyPrep DNA Gel Extraction Kit (Axygen Biosciences, Union City, CA, USA) according to the manufacturer’s instructions. Then, the library was paired-end sequenced using UPARSE version 7.1 and the sequence reads with 97% of sequence similarity were selected to build distinct operational taxonomic units (OTUs) [25]. The sparse curves of 16S rRNA gene sequences exhibit a tendency towards saturation platforms, ensuring a sufficient sequencing depth for diversity analysis. Analyses based on Mothur (version:1.21.1) revealed 97% of the same alpha diversity indices, including the Chao, ACE, Shannon and Simpson diversity indices. The Bray–Curtis distance was used to perform Principal Coordinate Analysis (PCoA). Linear discriminant effect size analysis (LEfSe) was used to compare the relative microbial community abundance between treatments [26].

### 2.10. Transcriptome Analysis of Ileal Tissue

The total RNA from ileal tissue was extracted with trizol reagent. Subsequently, the concentration and purity of the total RNA was detected with the RNA Nano 6000 Assay Kit and the Bioanalyzer 2100 system (Agilent, Santa Clara, CA, USA). A quantity of 1.0 μg of RNA per sample was used to create RNA-Seq libraries. The quality of the cDNA libraries was assessed using the Agilent Bioanalyzer 2100 system according to the manufacturer’s instructions. To ensure data quality, we filtered the raw data before analysis to exclude low-quality and invalid data, and obtain clean reads. We used feature counts (version 1.5.0-p3) to map each read to its corresponding gene and calculate the fragments per kilobase per million mapped fragments (FPKM) of each gene based on its length and read count. Differential expression analyses were performed using the DESeq2 R package (Version 1.20.0) after applying the screening criteria (*p* < 0.05; log |FC| > 1). Multiple testing was corrected using the Benjamini and Hochberg method. The Venn Diagram package (Version 3.0.3) was used to screen for the co-expression of differentially expressed genes. A cluster analysis (two-way clustering) of the FPKM values of the differential genes was performed using the pheatmap package (Version 3.0.3). KEGG pathway enrichment of the co-expressed differential genes was performed using the cluster Profiler R package (Version 3.0.3).

### 2.11. Short-Chain Fatty Acids (SCFAs) of Ileal Contents

Based on our previous study [27], the short-chain fatty acids (SCFAs) in ileal digests were quantified using a GC-14B gas chromatograph (Shimadzu, Kyoto, Japan). In short, approximately 0.5 g of ileum digesta was dissolved in double-distilled water (500 μL), shaken for 30 min, and then centrifuged at 12,000× *g* for 10 min. The extracted supernatant was mixed with metaphosphoric acid and crotonic acid at a ratio of 1:4 and stored at −20 °C overnight. The mixture was left at room temperature for 3 h before the assay. Following vortexing and centrifugation, the supernatant was filtered through a 0.6 µm filter and analyzed for SCFAs.

### 2.12. Statistical Analysis

Each animal was considered as the experimental unit for all responses measured (*n* = 6). All data were analyzed by SPSS 20.0 (SPSS Inc., Chicago, IL, USA) and expressed as means with standard error of the mean ± SEM. Statistical analysis was performed by one-way analysis of variance (ANOVA) followed by Tukey’s multiple comparison tests. All images were generated using GraphPad Prism 9.5 (GraphPad Software, Inc., La Jolla, CA, USA). * *p* < 0.05, ** *p* < 0.01, *** *p* < 0.001. Spearman correlation analysis was performed using the corrplot package in R software (Version 3.0.3), *p* < 0.05, and absolute r > 0.5 was considered to be significantly relevant.

## 3. Results

### 3.1. Effect of Cordyceps militaris Extract and Cordycepin on Growth Performance of Piglets before LPS Challenge

For the entire three-week duration of the experiment period preceding the LPS challenge, the initial BW, final BW, ADG, ADFI and F/G of the piglets are shown in Table 2. The ADG, ADFI and F/G of the piglets in the CON group, CPN group and CME group were not significantly different in terms of ADG, ADFI and F/G among the three groups (*p* > 0.05).

### 3.2. Cordyceps militaris Extract and Cordycepin Attenuates Intestinal Morphology Damage and Improves Intestinal Indices in LPS-Induced Piglets

**Figure 1:**
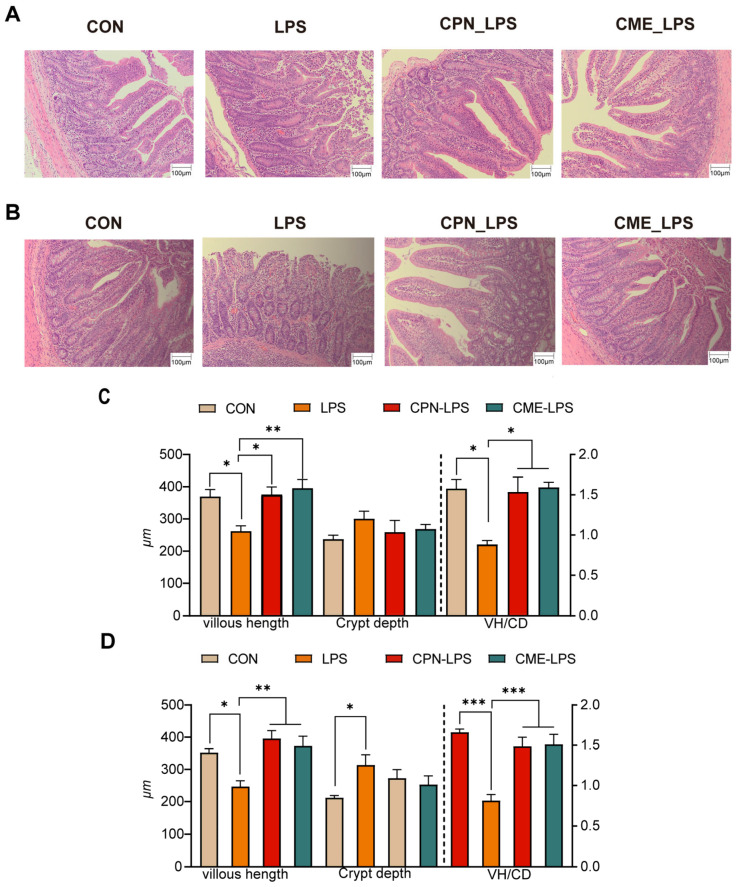
*Cordyceps militaris* extract and cordycepin attenuate intestinal morphology damage. (**A**) H&E-stained jejunal sections. (**B**) H&E-stained ileum sections. (**C**) The VH, CD, and VH/CD in the jejunal and (**D**) ileum were quantified. Scale bar, 100 μm. Data are expressed as mean ± SEM (*n* = 6). Statistical analysis was performed (one-way ANOVA followed by Tukey’s multiple comparisons tests) using SPSS software. * *p* < 0.05, ** *p* < 0.01 and *** *p* < 0.001.

The effects of *Cordyceps militaris* extract and cordycepin on the intestinal morphology of LPS-induced piglets are shown in Figure 1. The H&E-stained jejunal sections show that LPS changed the intestinal morphology of the piglets, leading to intestinal damage and intestinal hemorrhage compared to the CON group, which was alleviated in the CPN-LPS and CME-LPS groups (Figure 1A). In particular, LPS significantly reduced the villi height (*p* < 0.05) and villus height to crypt depth ratio (VH/CD) (*p* < 0.05), increased the jejunal crypt depth. Compared with the LPS group, the villi height and VH/CD values were significantly increased (*p* < 0.05), while the crypt depth was not significantly altered (*p* > 0.05) in the CPN-LPS and CME-LPS groups (Figure 1C). Notably, a similar phenomenon to that of the H&E-stained jejunal sections was observed in the HE-stained ileal sections. Compared to the CON group, the LPS group exhibited a significant disruption of the ileal mucosa and surface epithelium, severe hemorrhage, a significant decrease in the villus height (*p* < 0.05), and a significant increase in the crypt depth (*p* < 0.05). Additionally, the VH/CD was significantly decreased (*p* < 0.001) in the ileum, whereas the reversal of the shortening of the ileal villus length (*p* < 0.01) was observed in the CPN group and the restoration of VH/CD (*p* < 0.001) was observed in the CPN and CME treatments (Figure 1B,D). There were no significant differences in the gastrointestinal index and PH across the four groups (*p* > 0.05; Table 3). In conclusion, supplementation with *Cordyceps militaris* extract and cordycepin ameliorated the structural disruption of intestinal villi induced by LPS and maintained the intestinal morphostructural integrity.

### 3.3. Cordyceps militaris Extract and Cordycepin Relieved LPS-Induced Metabolic Disorder and Oxidative Stress

**Figure 2:**
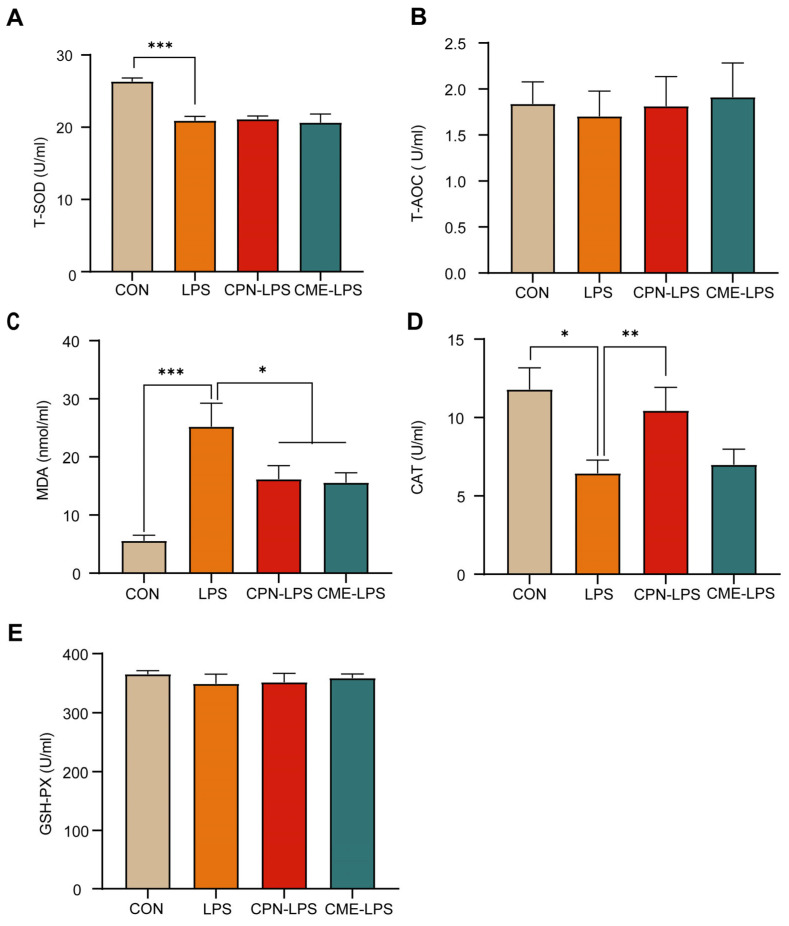
*Cordyceps militaris* extract and cordycepin relieved LPS-induced metabolic disorder and oxidative stress. (**A**) Serum T-SOD level. (**B**) Serum T-AOC level. (**C**) Serum MDA level. (**D**) Serum CAT level. (**E**) Serum T-AOC level. Data are expressed as mean ± SEM (*n* = 6). Statistical analysis was performed (one-way ANOVA followed by Tukey’s multiple comparisons tests) using SPSS software (Version 20.0). * *p* < 0.05, ** *p* < 0.01 and *** *p* < 0.001.

In this experiment, serum antioxidant indices (T-AOC, T-SOD, MDA, CAT and GSH-PX) were measured to assess the effects of CPN or CME on LPS-induced intestinal inflammation in piglets. As shown in Figure 2, LPS significantly decreased the levels of T-SOD (*p* < 0.001) and CAT (*p* < 0.05; Figure 2A,D), while significantly increasing the levels of MDA (*p* < 0.001) compared to the CON group; meanwhile, the CPN and the CME prevented the increase in MDA by LPS injection (*p* < 0.05; Figure 2C). In addition, the CPN treatment prevented a LPS-induced decrease in the CAT level (*p* < 0.01). In contrast, the CME treatment did not affect the CAT content (Figure 2D). There were no significant differences in T-AOC and GSH-PX across the four groups (*p* > 0.05; Figure 2B,E).

**Figure 3:**
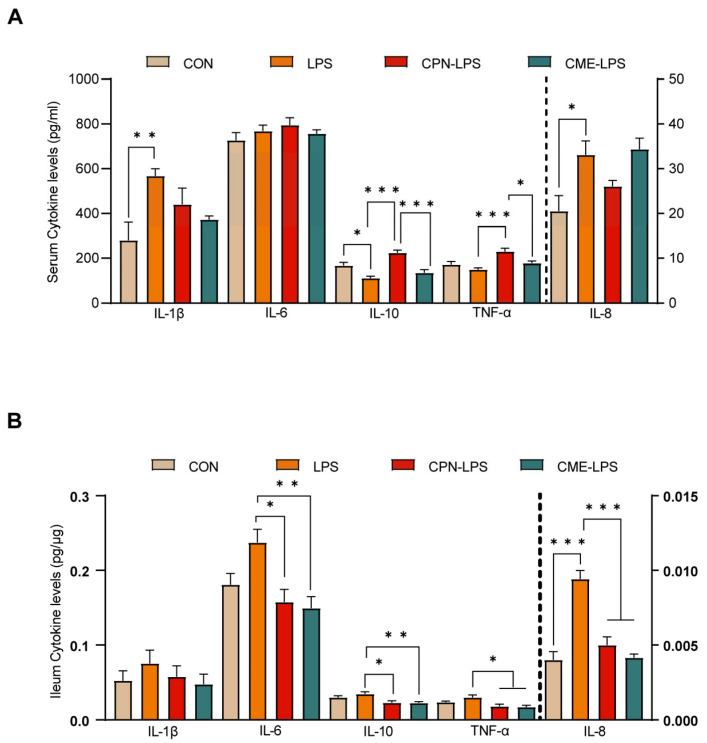
*Cordyceps militaris* extract and cordycepin attenuated the inflammatory response in LPS-induced piglets. (**A**) Serum cytokine levels. (**B**) Ileum cytokine levels. Data are expressed as mean ± SEM (*n* = 6). Statistical analysis was performed (one-way ANOVA followed by Tukey’s multiple comparisons tests) using SPSS software. * *p* < 0.05, ** *p* < 0.01 and *** *p* < 0.001.

The stimulation of LPS leads to strong immunostimulatory effects, inducing an inflammatory response. The serum and ileal mucosal cytokine levels were measured to evaluate the effect of the two different treatments on the inflammatory response to LPS challenge. The cytokine concentrations are shown in Figure 3. We observed that LPS induced a significant increase in serum cytokine IL-1β and IL-8 levels, as well as a significant decrease in IL-10 levels compared to the CON group (*p* < 0.05). The CPN treatment reversed the decrease in IL-10 levels induced by LPS challenge (*p* < 0.05), but also increased the TNF-α levels (*p* < 0.05) compared to the LPS group. Furthermore, the CPN treatment had the same differential expression results as the LPS group compared to the CME-LPS group (Figure 3A). The measurement of ileal mucosal cytokines revealed that IL-8 levels were increased in the LPS treatment group compared to the CON group (*p* < 0.05). Additionally, the levels of IL-6, IL-10, TNF-α, and IL-8 were significantly decreased in both the CPN-LPS and CME-LPS groups compared to the LPS group (*p* < 0.05; Figure 3B). Collectively, the results presented above indicate that *Cordyceps militaris* extract and cordycepin attenuate oxidative stress and the inflammatory response in LPS-induced piglets.

### 3.4. Cordyceps militaris Extract and Cordycepin Altered the Microbial Composition and SCFAs of the Ileum in LPS-Induced Piglets

**Figure 4:**
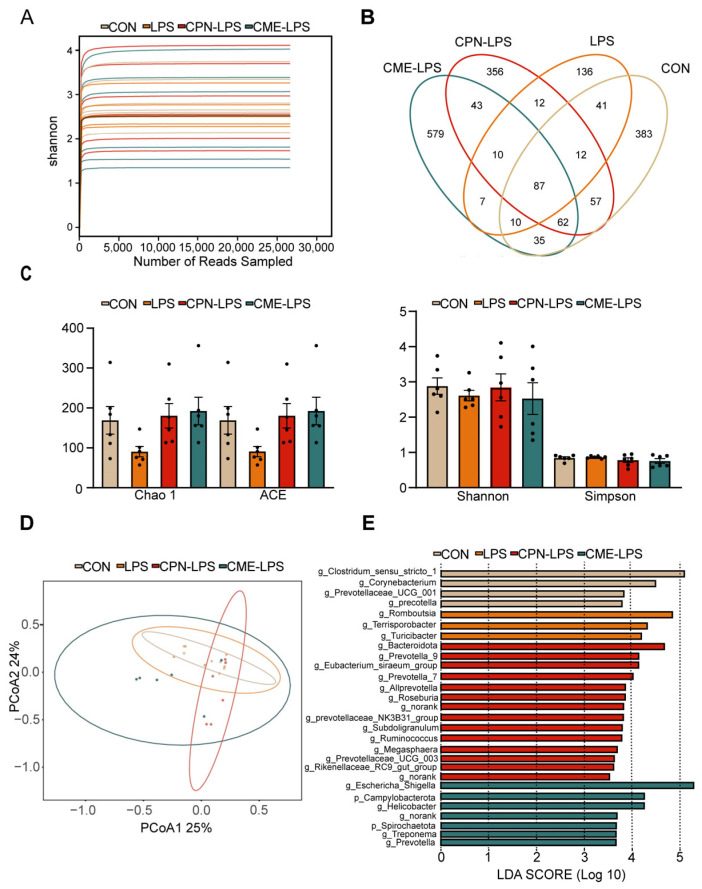
*Cordyceps militaris* extract and cordycepin altered the microbial composition of the ileum in LPS-induced piglets. (**A**) Shannon–Wiener rarefaction curve. (**B**) Venn diagram of OTUs in ileal chyme. (**C**) Alpha diversity of colonic microbiota of different groups. Statistical analysis was performed (one-way ANOVA followed by Tukey’s multiple comparisons tests) using SPSS software. (**D**) PCoA plot based on Bray–Curtis algorithm at the OUT level. (**E**) The LEfSe analysis (LDA score ≥ 2, *p* < 0.05). Data are expressed as mean ± SEM (*n* = 6).

To determine the composition of the ileal microbiota, 16sRNA gene amplicon sequencing analysis was performed. The Shannon–Wiener index illustrates the microbial diversity index by sequencing samples at different depths (Figure 4A). Moreover, the Venn plot shows that the specific OTUs for the CON group, LPS group, CPN-LPS group and CME-LPS group are 383, 136, 356, 579, respectively (Figure 4B). Furthermore, the richness and diversity of the microbial communities of the samples were reflected by Alpha diversity analysis. There were no significant differences in the richness and diversity indices, including the Chao 1 index, ACE index, Shannon index and Simpson index, among the four treatments (*p* > 0.05; Figure 4C). The Bray–Curtis algorithm-based PCoA (Figure 4D) revealed that the first principal component (PCoA1) and the second principal component (PCoA2) explained 25.00% and 24.00% of the variation in the microbial diversity, respectively. Meanwhile, the LEfSe analyses and LDA score showed significant differences in species abundance at the phylum level and genus level across groups (Figure 4E). There were 28 discriminative species identified among the four treatments in the ileal digesta of piglets. *Clostridium*_sensu_stricto_1, *Corynebacterium*, Prevotellaceae_UCG_001 and *Prevotella* were enriched in the CON group; *Romboutsia*, *Terrisporobacter* and *Turlclbacter* were enriched in the LPS group; *Bacteroidota*, *Prevotella*_9, *Prevotella*_7 and *Allprevotella* were enriched in the CPN-LPS group; and *Campylobacterota*, *Spirochaetota*, *Hellcobacter* and *Prevotella*_9 were significantly enriched in the CME-LPS group.

**Figure 5:**
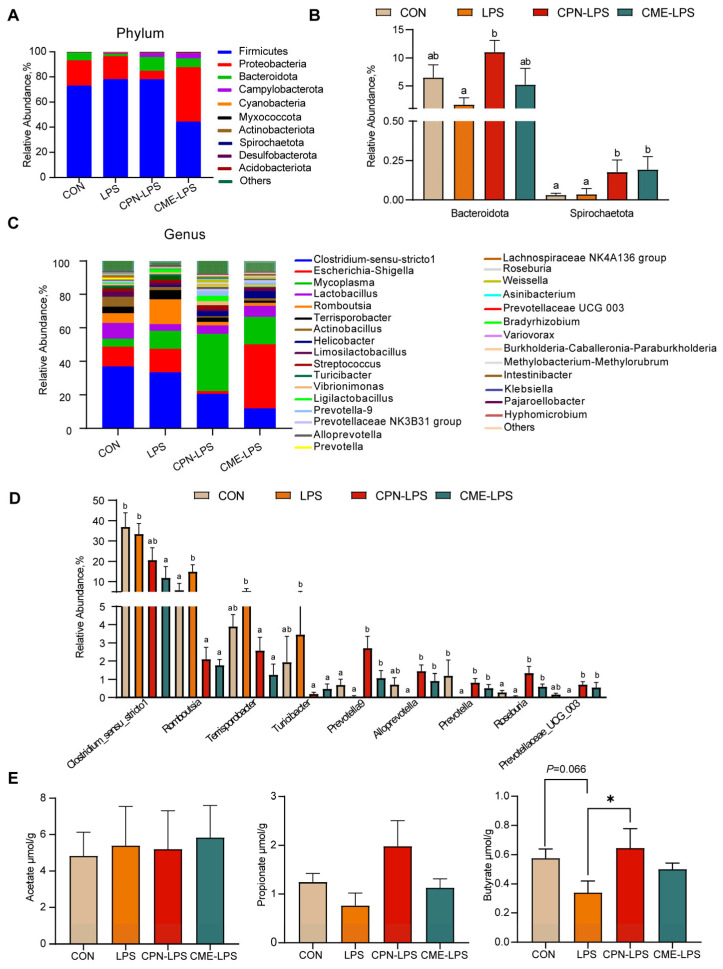
The effect of *Cordyceps militaris* extract and cordycepin on the relative abundance of ileal microbiota at different levels and the content of SCFAs in the ileum. (**A**) Taxonomic composition of ileum contents of piglets at phylum levels. (**B**) The significant changes in the differential ileum microbiota at the phylum level. Statistical analysis by Kruskal–Wallis sum-rank test and Wilcoxon rank-sum test. Different letters represent significant differences (*p* < 0.05). (**C**) Taxonomic composition of ileum contents of piglets at the genus level. (**D**) The significant changes in the differential ileum microbiota at the genus level. Statistical analysis by Kruskal–Wallis sum-rank test and Wilcoxon rank-sum test. Different letters represent significant differences (*p* < 0.05). (**E**) Concentration of acetate, propionate and butyrate in the contents of the ileum. Data are expressed as mean ± SEM (*n* = 6). Statistical analysis was performed (one-way ANOVA followed by Tukey’s multiple comparisons tests) using SPSS software. * *p* < 0.05.

The results of the further analysis of the differential species and microbiota structure are shown in Figure 5. The relative abundance of different phylum levels shows that the dominant bacterial groups were Firmicutes, Proteobacteria, Bacteroidota and Campylobacterota (Figure 5A). LPS reduced the relative abundance of *Bacteroides* compared with the CON group (*p* < 0.1), and the treatment of CPN alleviated the LPS-induced challenge and increased the abundance of *Bacteroides* (*p* < 0.05). Compared to the CON group and LPS group, the treatment of CME and CPN significantly increased the relative abundance of *Spirochaetota* (*p* < 0.05; Figure 5B). Among the top 30 genera in terms of relative abundance, compared to the CON group, LPS significantly increased the relative abundances of *Romboutsia* (*p* < 0.05) and significantly decreased the relative abundances of *Prevotella* (*p* < 0.05); the relative abundance of Prevotellaceae UCG-003 shows a decreasing trend (*p* < 0.1), whereas supplementation with CPN and CME attenuated LPS-induced increases in *Romboutsia* and decreases in *Prevotella* and Prevotellaceae UCG-003 (*p* < 0.05; Figure 5C,D). Furthermore, the relative abundance of *Terrisporobacter* and *Turicibacter* was significantly reduced (*p* < 0.05), while the relative abundance of *Prevotella*-9, *Alloprevotella* and *Roseburia* was significantly increased (*p* < 0.05) in piglets from the CPN-LPS and CME-LPS groups compared to those from the LPS group. Additionally, the relative abundance of *Prevotella*-9 and *Roseburia* was significantly upregulated in the CPN treatment group compared to the control group (*p* < 0.05). Nevertheless, the CME treatment significantly downregulated the relative abundance of *Clostridium*-msensu-stricto1 compared to the CON and LPS groups (*p* < 0.05). Strikingly, compared to the CPN-LPS group, the relative abundance of *Escherichia-Shigella* was significantly increased in the CME-LPS group (*p* < 0.01). Taken together, CPN and CME differentially modified the gut microbiota.

To evaluate the effect of the two distinct treatments on gut microbial metabolites, we quantified the concentrations of acetate, propionate and butyrate in the ileal contents. As shown in Figure 5, compared to the CON group, LPS reduced the trend in the butyrate content (0.05 < *p* < 0.1), whereas the CPN treatment reversed the reduction in the butyrate content caused by LPS (*p* < 0.05; Figure 5E). There were no significant differences in the content of SCFAs in the other groups (*p* > 0.05).

### 3.5. Transcriptomics Profiling of Ileum Tissue

**Figure 6:**
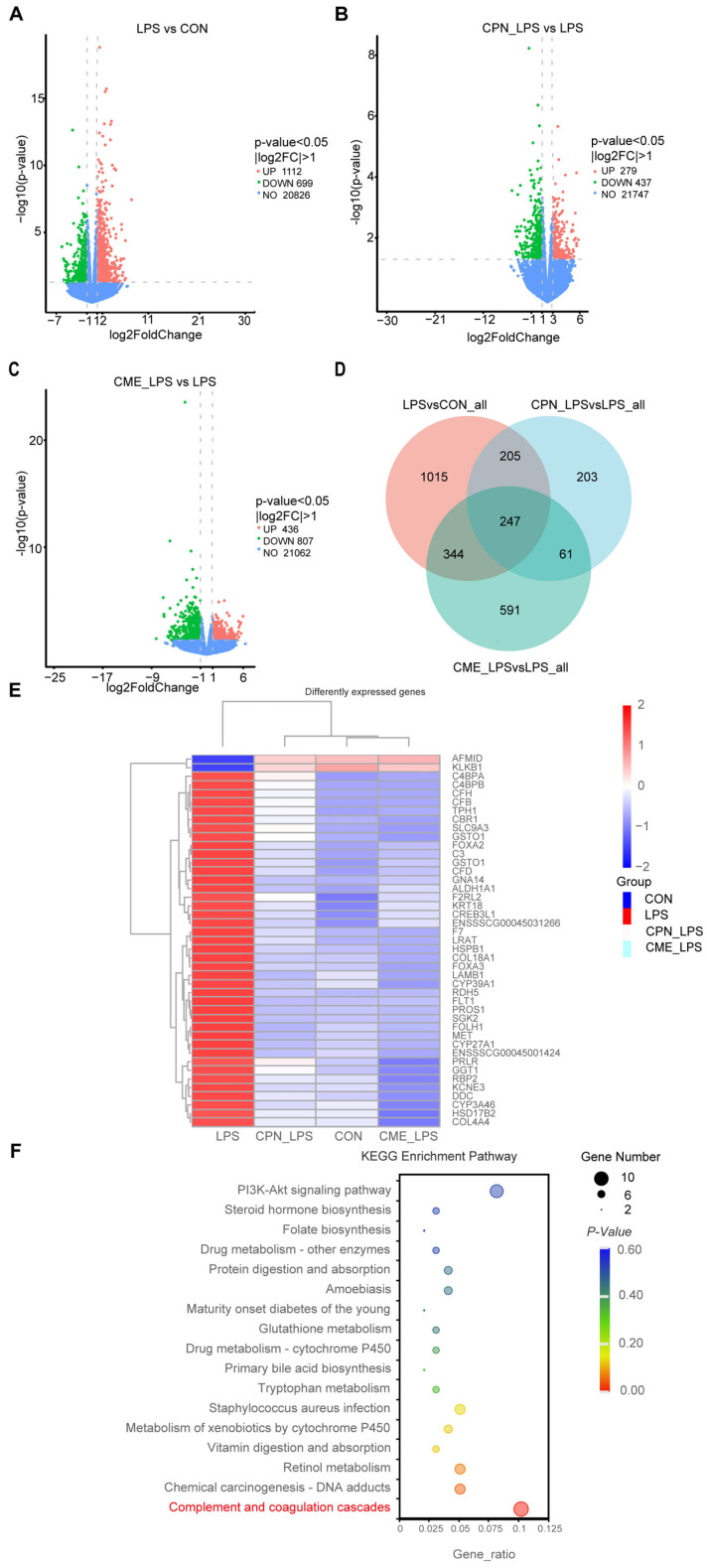
Alterations in the transcriptome of ileal tissues by *Cordyceps militaris* extract and cordycepin (*n* = 3). Volcano map of differentially expressed gene distribution between the LPS group and CON group (**A**), the CPN-LPS group and LPS group (**B**), and the CME-LPS group and LPS group (**C**). (**D**) Venn diagram of co-expressed genes. (**E**) Heat clustering map of the 43 DEGs. (**F**) KEGG enrichment analysis.

The above experiments demonstrated that the CPN and CME treatments had a mitigating effect on LPS-induced intestinal inflammation in piglets. Differentially expressed genes (DEGs) were selected based their on *p*-value < 0.05 and |log2FoldChange| > 1. According to the volcano plot showed, a total of 1811 genes were identified as differentially expressed between the CON and LPS groups (Figure 6A). Of these, 1112 genes were up-regulated and 699 genes were downregulated. Additionally, a total of 716 genes were identified as differentially expressed between the CPN-LPS and the LPS groups, with 279 genes upregulated and 437 genes downregulated (Figure 6B). The CME-LPS and LPS groups had a total of 1243 DEGs, with 436 genes upregulated and 807 genes downregulated (Figure 6C). The Venn diagram further screened 247 co-regulated genes (including 21 unknown genes) that were altered among the groups, which could be important in the treatment of bowel inflammation (Figure 6D). Of these, the heatmap generated from clustering showed 43 DEGs that were closely related to inflammation and phagocytosis (Figure 6E). Furthermore, we performed a Kyoto Encyclopedia of Genes and Genomes (KEGG) pathway enrichment analysis of the above DEGs to identify potential drug targets, encompassing 17 significantly differentiated pathways (*p* < 0.05). DEGs were mainly enriched in the “Complement and coagulation cascade”, “Staphylococcus aureus infection” and “PI3K-AKT signaling pathway” (Figure 6F). LPS affected the complement and coagulant pathways, upregulating nine DEGs (*CFD*, *F2RL2*, *CFB*, *C4BPA*, *F7*, *C4BPB*, *CFH*, *C3*, *PROS1*) and downregulating *KLKB1* compared to the CON group. However, the CPN-LPS group and the CME-LPS group reversed the LPS-induced upregulation of DEGs and restored the level of KLKB1 (Table 4). Therefore, we speculate that CME and CPN may regulate intestinal immunity through the “complement and coagulation cascade” signalling pathway.

### 3.6. Correlation Analysis of Ileal Microbiota with Transcriptional and Biochemical Indices

**Figure 7:**
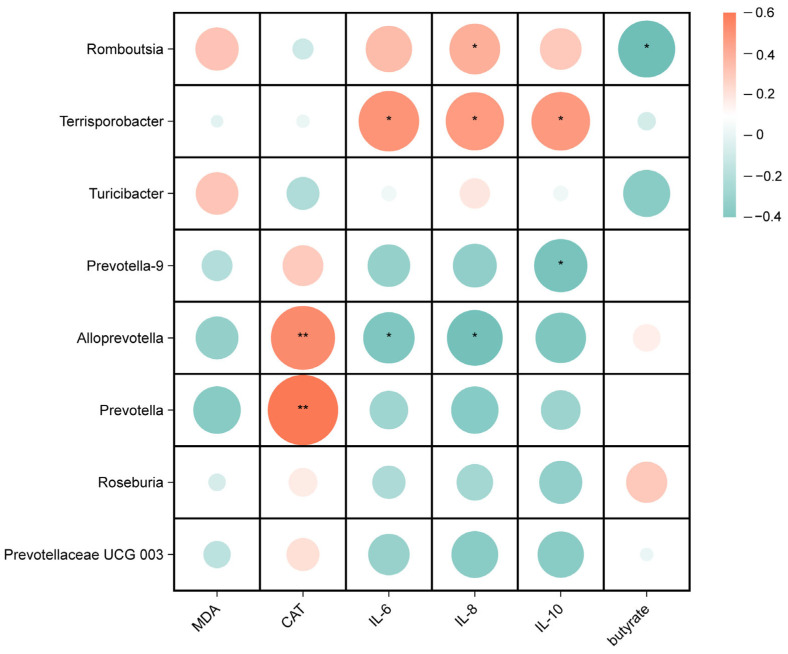
Correlation analysis between ileal microbiota and serum antioxidant and inflammation indicators, and butyrate. * *p* < 0.05, ** *p* < 0.01.

Spearman correlation analyses were performed to investigate the potential associations between gut microbes and biochemical parameters in weaned piglets, including oxidative indicators, inflammatory factors, and SCFAs. As shown in Figure 7, for the microorganisms of the ileal digesta genus, the relative abundance of *Romboutsia* was positively correlated with IL-8 (*p* < 0.05) and negatively correlated with the butyrate content (*p* < 0.05), the relative abundance of *Terrisporobacter* was positively correlated with IL-6, IL-8, and IL-10 (*p* < 0.05), the relative abundances of *Alloprevotella* and *Prevotella* were positively correlated with CAT (*p* < 0.01), and the relative abundance of *Alloprevotella* was negatively correlated with IL-6 and IL-8 (*p* < 0.05).

## 4. Discussion

Immune system dysfunction and the invasion of pathogenic microorganisms during the weaning period usually cause weaning stress, which reduces the growth performance of piglets, disrupts the intestinal microbiota, and causes intestinal dysfunction, diarrhea and even death [3,28]. Natural bioactive substance supplements can be an effective solution to alleviating weaning stress in piglets [29]. In this study, we established an LPS-induced intestinal injury model in piglets. *Cordyceps militaris* is known for its active bioactive components and has been extensively researched for its various biological activities, such as its anti-inflammatory and antioxidative effects and ability to attenuate intestinal damage [30]. To date, there has been limited research on the use of supplementation with *Cordyceps militaris* extract and cordycepin to modulate LPS-induced oxidative stress and intestinal inflammation in piglets and its relationship with changes in the intestinal microbiota. In our study, it was demonstrated that the addition of CME and CPN significantly improved immune regulation and oxidative stress in LPS-induced piglets. No significant effects regarding the growth performance of piglets before LPS induction were observed compared to the CON group, which was consistent with previous studies [31]. Therefore, we hypothesized that this may be related to the low concentration of supplemental CPN and CME. Additionally, the intestinal microbiota was affected by supplementation with CME and CPN, which promoted microbial metabolites and maintained a stable intestinal environment. Collectively, our work provides evidence that CME and CPN have the potential to serve as a viable nutritional intervention to mitigate intestinal damage in piglets.

Intestinal morphological and functional integrity is a key indicator of nutrient absorption and gut health [24]. *Cordyceps militaris* powder prophylactic supplementation alleviated intestinal mucosal barrier damage and attenuated the intestinal histological pathology in DSS-induced UC mice [32]. In the present experiment, we observed that LPS induction significantly reduced the villus height and villus height to crypt depth ratio after the intraperitoneal injection of LPS, whereas the CPN and CME treatments attenuated the damage to the intestinal morphology, which is consistent with previous reports [8,33]. Interestingly, the CME treatment showed better therapeutic effects in the jejunum compared to the CPN treatment.

LPS-induced oxidative stress in the intestine may be the cause of damage to the intestinal structure [22]. Our results demonstrated that LPS provoked a significant increase in the serum MDA concentration and decreased the activities of T-SOD and CAT compared to the CON group. MDA is one of the metabolites of lipid peroxidation and can be used to indicate the oxidative stress status [34,35]. SOD and CAT are crucial antioxidants and free radical scavengers in the body [36]. This study indicates that LPS exposure caused oxidative stress in piglets. Supplementation with CPN or CME significantly reduced the elevated MDA concentration induced by LPS challenge. Additionally, CAT activity was increased in the CPN group of piglets. Overall, our results prove that supplementation with CME or CPN has a protective effect, alleviating oxidative stress in weaned piglets.

LPS stimulates the immune system, causes an inflammatory response in the body, alters the ratio of pro-inflammatory cytokines to anti-inflammatory factors in the gut and thus exacerbates intestinal inflammation [37]. C-reactive protein (CRP) and haptoglobin (HP) are acute phase proteins synthesized by hepatocytes. CRP responds to increases in peripheral pro-inflammatory cytokines, such as TNF-α and IL-6. Serum acute-phase protein levels are a useful clinical indicator of systemic pro-inflammatory activity in various inflammatory conditions [38,39]. The current experiment aimed to investigate the impact of LPS induction on intestinal inflammation in piglets. Therefore, we chose the same inflammatory indicators from similar studies to assess the effect of LPS induction on the intestinal inflammatory response in piglets [1,24]. The overexpression of pro-inflammatory cytokines (TNF-α, IL-1β, IL-6 and IL-8) induces intestinal mucosal damage, alters intestinal epithelial permeability and triggers an inflammatory response [40]. In the present experiment, the supplementation of CPN or CME prevented the reduction in serum IL-10 levels caused by LPS induction. However, the CPN-LPS group showed elevated serum TNF-α levels, which was inconsistent with the data mentioned above from the previous studies [8]. In the ileum, both treatments significantly reduced the TNF-α, IL-6, IL-8 and IL-10 levels. The increase in serum TNF-α levels and decrease in ileal TNF-α levels in the CPN-LPS group may be attributed to the fact that TNF-α alters the integrity of the intestinal epithelial cells and thus enhances intestinal permeability [41]. The reduction in IL-10 may be related to the controversial nature of its role in the intestine [42]. Taken together, the results indicate that interventions involving CPN or CME supplementation can relieve intestinal mucosal injury by inhibiting the overproduction of pro-inflammatory cytokines and promoting the secretion of anti-inflammatory factors.

The intestinal microbiota and its metabolites are essential for intestinal function and health, and that they are involved in nutrient digestion and immunomodulation in the host [31,43]. An early study reported that *Cordyceps militaris* altered the colonic microbial composition in a porcine model [31]. Our data revealed that the two different therapeutic treatments altered the composition and structure of the gut microbiota and increased the number of OTUs, but did not significantly alter α-diversity, which may be because of the shorter duration of LPS induction. Studies have shown that Firmicutes, Bacteroidota, Actinobacteria, and Proteobacteria are the main dominant bacterial phylum in the intestinal tract of healthy piglets [2]. The relative abundance of Bacteroidetes was significantly reduced in LPS-induced acute colitis mice [44]. In the present study, the relative abundance of Bacteroidota was reduced in the LPS group compared to the CON group. Compared to the LPS group, the CPN treatment reversed the LPS-induced decrease in the relative abundance of Bacteroidota, and the treatment with CME and CPN increased the relative abundance of Spirochaetota. This increased relative abundance of Bacteroidota contributes to the maintenance of intestinal health and the alleviation of intestinal inflammation and LPS-induced systemic inflammation [45,46]. Previous studies have suggested that the supplementation of broiler chickens with fermented herbal residues (FCMRs) increases the relative abundance of cecal Spirochaetota, which facilitates the conversion of carbohydrates to volatile fatty acids (VFAs) to provide energy to the body [47,48]. In conclusion, the addition of CME and CPN prevented the decrease in the relative abundance of beneficial bacteria caused by LPS induction and increased the number of acid-producing bacteria.

More importantly, we found that LPS induction decreased the relative abundance of *Prevotella* UCG-003 and *Prevotella* and increased the relative abundance of *Romboutsia* compared to the CON group; both distinct treatment groups restore the relative abundance of SCFA-producing bacteria such as *Prevotella* UCG-003 and *Prevotella* [49,50]. *Romboutsia* is considered to be an intestinal pathogen that causes inflammation, and it has been reported to inhibit butyrate production at elevated levels [51]. Furthermore, the relative abundance of *Terrisporobacter* and *Turicibacter* was reduced, while the relative abundances of *Prevotella*-9, *Alloprevotella*, and *Roseburia* increased in piglets in the CPN-LPS and CME-LPS groups compared to the LPS group. An increase in the relative abundance of *Terrisporobacter* and *Turicibacter* has been shown to positively correlate with the mRNA expression of pro-inflammatory factors (IL-1β), which in turn induce the development of colitis in mice [52,53,54]. *Roseburia*, a butyrate-producing probiotic, could suppress intestinal inflammation through metabolites [55]. Notably, our study observed that compared to the CON and LPS groups, the CME-LPS group had a decreased relative abundance of *Clostridium*-msensu-stricto1. *Clostridium*-msensu-stricto1 colonizes piglets early in development and increased levels of it are associated with intestinal inflammation. This substance has the potential to cause necrotizing enterocolitis [56,57]. In conclusion, both CPN and CME modulated intestinal flora structures, promoted the abundance of beneficial bacteria and inhibited harmful bacteria, thus significantly improving the degree of intestinal damage and alleviating intestinal inflammation in an LPS-induced piglet model.

SCFAs are crucial for maintaining the integrity and function of the intestinal tract [41]. Butyrate supplementation can reduce LPS challenge-induced gut damage and inflammation, resulting in lower serum LPS and IL-6 levels [58]. However, previous reports have shown that supplementation with *Cordyceps militaris* increases acetate and butyrate production in both pig and mouse models [31,59]. In this experiment, the CPN treatment prevented the decrease in the butyrate concentration caused by LPS. This further demonstrated that increasing the content of short-chain fatty acids may be an effective way for cordycepin to provide intestinal protection.

Transcriptomics was used to investigate the potential pathways involved in the mitigation of intestinal damage by *Cordyceps militaris* extract during LPS induction. In the present study, we screened for differentially expressed genes among various groups using a volcano plot. A total of 247 co-expressed genes were screened using a co-expression Venn plot. Genes with significant differences were analyzed further and enriched using KEGG pathway analysis. Analysis of the DEGs through the KEGG pathway database revealed that they were primarily enriched in pathways including “complement and coagulation cascades”, the “PI3K-Akt pathway signaling pathway”, “Staphylococcus aureus infection”, “Glutathione metabolism” and “Primary bile acid biosynthesis”. Most of these pathways are associated with the immune system and infectious diseases [60,61,62,63]. This study showed that LPS upregulated the expression of differentially expressed genes involved in complement and coagulation cascade reactions, including CFD, F2RL2, CFB, C4BPA, F7, C4BPB, CFH, C3 and PROS1, and downregulated KLKB1 levels. In contrast, supplementation with CPN or CME was able to modify the upregulation of differentially expressed genes and restore KLKB1 levels following LPS treatment. The above results have demonstrated that the aberrant activation of the complement system can result in the onset of various illnesses, and that the upregulation of the pro-inflammatory complement factors CFD and CFB induces an inflammatory response [64,65]. Inflammatory infiltrating cells expressing high levels of CFH inhibit C3 depletion, promoting complement activation; meanwhile, CFH-deficient mice reduce macrophage atherosclerotic necrotic core areas [66]. Furthermore, C3 is involved in the regulation of the staphylococcus aureus infection pathway, which subsequently triggers an acute inflammatory response in both human and animal bodies [67]. C4BPA expression was found to be increased in patients with irritable bowel syndrome [68], while C4BPB expression was found to be increased in patients with inflammatory bowel disease [69]. In this study, the potential pathway of action able to mitigate the induction of LPS was found to possibly involve the alteration of genes in the complement and coagulation cascades through CPN or CME treatment.

*Romboutsia* is considered to be an intestinal pathogen that causes inflammation, and has been reported to inhibit butyrate production at elevated levels [51]; this is consistent with our results. *Terrisporobacter* was found to be positively correlated with *IL-1β* mRNA expression and LPS content, and was negatively correlated with relative *IL-10* mRNA expression levels [54]. Correlation analysis revealed a positive correlation between the relative abundances of *Terrisporobacter* and the pro-inflammatory factors IL-6 and IL-8. This further supports the potential involvement of *Terrisporobacter* in IBD [70]. Kuijieyuan Decoction alleviates the decrease in the relative abundance of *Alloprevotella* and *Prevotella* and the decrease in CAT activity in ulcerative colitis rats [71]. The present study also demonstrates that the relative abundances of *Alloprevotella* and *Prevotella* were positively correlated with CAT. Therefore, this study indicates that supplementation with CME or CPN can modify the structure of the intestinal microbiota and increase the abundance of beneficial gut bacteria in LPS piglets. Simultaneously, it alters the concentration of short-chain fatty acids in the intestine, relieves oxidative stress and reduces the inflammatory response. These data indicate that CME or CPN supplementation reduces LPS-induced intestinal damage and maintains intestinal health.

## 5. Conclusions

In conclusion, our findings demonstrate that dietary CPN or CME supplementation could alleviate intestinal mucosal barrier damage and suppress the intestinal inflammation and oxidative stress caused by LPS-induced intestinal inflammation in piglets. CPN or CME interventions maintain the integrity of the gut barrier by modulating the structure of the gut microbiota and increasing microbial metabolite production. In addition, complement and coagulation cascade responses may be potential pathways that prevent LPS-induced intestinal injury. This study offers a novel approach to utilizing CPN or CME as a functional feed additive to improve intestinal health through nutritional intervention in piglets.

## Figures and Tables

**Table 1:** Composition and nutrient level of the basal diet (as fed basis).

Ingredient	Percentage (%)	Calculated Nutritional Compositions (%)	
Corn	70.0	Digestive energy (MJ/kg)	14.60
Soybean meal	18.0	Crude protein	16.00
Wheat bran	6.50	Lysine	1.23
Soybean oil	1.90	Methionine + Cystine	0.70
Lysine	0.69	Threonine	0.79
Methionine	0.24	Tryptophan	0.22
Threonine	0.30		
Tryptophan	0.07		
Calcium hydrogen phosphate	0.45		
Stone powder	0.50		
Salt	0.30		
Multivitamins ^1^	0.03		
Minerals ^2^	0.20		
Choline chloride (50%)	0.12		
Zeolite powder	0.60		
Antioxidant	0.1		
Total	100.0		

^1^ The mineral supply per kg of diet was as follows: Fe 165 mg, Zn 165 mg, Cu 16.5 mg, Mn 30 mg, Co 0.15 mg, I 0.25 mg, Se 0.25 mg. ^2^ The multivitamin supply per kg of diet was as follows: VA 11 000 IU, VD3 1 000 IU, VE 16 IU, VK1 1 mg, VB1 0.6 mg, VB2 0.6 mg, d-pantothenic acid 6 mg, nicotinic acid 10 mg, VB12 0.03 mg, folic acid 0.8 mg, VB6 1.5 mg.

**Table 2:** Effects of different *Cordyceps militaris* extracts and cordycepin on the growth performance of weaned piglets.

Treatment	CON	CPN	CME	*p*-Value
Initial BW/kg	7.33 ± 0.29	7.20 ± 0.19	7.33 ± 0.07	0.89
Final BW/kg	13.80 ± 0.93	13.47 ± 0.77	13.67 ± 0.87	0.96
ADFI/g	529.33 ± 53.00	536.33 ± 53.34	517.17 ± 49.19	0.96
ADG/g	308.33 ± 36.92	300.00 ± 29.89	301.67 ± 39.53	0.99
F/G	1.74 ± 0.05	1.82 ± 0.12	1.76 ± 0.08	0.85

CON = Control group; CPN = Cordycepin group; CME = *Cordyceps militaris* extract group. Data were expressed as mean ± SEM (*n* = 6). Different letters represent significant differences (*p* < 0.05) by one-way ANOVA followed by Tukey’s multiple comparisons tests.

**Table 3:** Effects of *Cordyceps militaris* extract and cordycepin on gastrointestinal indices of LPS-induced piglets.

Treatment	CON	LPS	CPN-LPS	CME-LPS	*p*-Value
Intestine indices, %					
Stomach	1.48 ± 0.34	2.04 ± 0.25	1.76 ± 0.17	1.56 ± 0.12	0.39
Jejunum	5.86 ± 0.42	5.59 ± 0.38	5.14 ± 0.46	5.26 ± 0.35	0.59
Ileum	0.15 ± 0.01	0.17 ± 0.02	0.14 ± 0.01	0.16 ± 0.01	0.42
Colon	2.81 ± 0.27 ^b^	2.18 ± 0.27 ^ab^	1.38 ± 0.27 ^ab^	1.37 ± 0.12 ^a^	<0.01
Gastrointestinal tract pH					
Stomach	4.51 ± 0.61	5.17 ± 0.26	5.08 ± 0.35	5.37 ± 0.50	0.57
Jejunum	6.68 ± 0.13	6.55 ± 0.24	6.73 ± 0.15	6.35 ± 0.09	0.37
Ileum	7.21 ± 0.08	7.47 ± 0.14	7.06 ± 0.27	7.18 ± 0.09	0.27
Colon	6.46 ± 0.08	6.43 ± 0.12	6.413 ± 0.12	6.63 ± 0.18	0.63

^a^: CON = Control; LPS = Lipopolysaccharide; CPN-LPS = Cordycepin + Lipopolysaccharide; CME-LPS = *Cordyceps militaris* extract + Lipopolysaccharide. Data are expressed as mean ± SEM (*n* = 6). Different letters represent significant differences (*p* < 0.05) by one-way ANOVA followed by Tukey’s multiple comparisons tests. ^b^: Intestine indices refer to the ratio of intestinal organ weight to the final body weight.

**Table 4:** LPS-induced differential genes expressed in the complement and coagulation cascade pathway in piglets.

Gene	Regulation			Description
	LPS vs. CON	CPN-LPS vs. LPS	CME-LPS vs. LPS	
*CFD*	Upregulated	Downregulated	Downregulated	Complement factor D
*F2RL2*	Upregulated	Downregulated	Downregulated	Coagulation factor II thrombin receptor like 2
*CFB*	Upregulated	Downregulated	Downregulated	Complement factor B
*C4BPA*	Upregulated	Downregulated	Downregulated	Complement component 4 binding protein alpha
*F7*	Upregulated	Downregulated	Downregulated	Coagulation factor VII
*C4BPB*	Upregulated	Downregulated	Downregulated	Complement component 4 binding protein β
*CFH*	Upregulated	Downregulated	Downregulated	Complement factor H
*C3*	Upregulated	Downregulated	Downregulated	Complement C3
*KLKB1*	Downregulated	Upregulated	Upregulated	Kinin releasing enzyme B1
*PROS1*	Upregulated	Downregulated	Downregulated	Protein S

CON = Control; LPS = Lipopolysaccharide; CPN-LPS = Cordycepin + Lipopolysaccharide; CME-LPS = *Cordyceps militaris* extract + Lipopolysaccharide (*n* = 3).

## Data Availability

Data are contained within the article.
